# Evaluation of patient DVH‐based QA metrics for prostate VMAT: correlation between accuracy of estimated 3D patient dose and magnitude of MLC misalignment

**DOI:** 10.1120/jacmp.v16i3.5251

**Published:** 2015-05-08

**Authors:** Noriyuki Kadoya, Masahide Saito, Makoto Ogasawara, Yukio Fujita, Kengo Ito, Kiyokazu Sato, Kazuma Kishi, Suguru Dobashi, Ken Takeda, Keiichi Jingu

**Affiliations:** ^1^ Department of Radiation Oncology Tohoku University School of Medicine Sendai Miyagi Japan; ^2^ Department of Radiation Oncology Tokai University School of Medicine Hachioji Tokyo Japan; ^3^ Radiation Technology Tohoku University Hospital Sendai Miyagi Japan; ^4^ Department of Radiological Technology School of Health Sciences, Faculty of Medicine, Tohoku University Sendai Miyagi Japan

**Keywords:** patient QA, radiotherapy, 3D patient dose, dose distribution comparison, diode array

## Abstract

The purpose of this study was to evaluate the accuracy of commercially available software, using patient DVH‐based QA metrics, by investigating the correlation between estimated 3D patient dose and magnitude of MLC misalignments. We tested 3DVH software with an ArcCHECK. Two different calculating modes of ArcCHECK Planned Dose Perturbation (ACPDP) were used: “Normal Sensitivity” and “High Sensitivity”. Ten prostate cancer patients treated with hypofractionated VMAT (67.6 Gy/26 Fr) in our hospital were studied. For the baseline plan, we induced MLC errors (−0.75,−0.5,−0.25,0.25,0.5, and 0.75 mm for each single bank). We calculated the dose differences between the ACPDP dose with error and TPS dose with error using gamma passing rates and using DVH‐based QA metrics. The correlations between dose estimation error and MLC position error varied with each structure and metric. A comparison using 1%/1 mm gamma index showed that the larger was the MLC error‐induced, the worse were the gamma passing rates. Slopes of linear fit to dose estimation error versus MLC position error for mean dose and D95 to the PTV were 1.76 and $1.40\%\;{\text{mm}}^{-1}$, respectively, for “Normal Sensitivity”, and −0.53 and $0.88\%\;{\text{mm}}^{-1}$, respectively, for “High Sensitivity”, showing better accuracy for “High Sensitivity” than “Normal Sensitivity”. On the other hand, the slopes for mean dose to the rectum and bladder, V35 to the rectum and bladder and V55 to the rectum and bladder, were −1.00,−0.55,−2.56,−1.25,−3.53, and $1.85\%\;{\text{mm}}^{-1}$, respectively, for “Normal Sensitivity”, and −2.89,−2.39,−4.54,−3.12,−6.24, and $-4.11\%\;{\text{mm}}^{-1}$, respectively, for “High Sensitivity”, showing significant better accuracy for “Normal Sensitivity” than “High Sensitivity”. Our results showed that 3DVH had some residual error for both sensitivities. Furthermore, we found that “Normal Sensitivity” might have better accuracy for the DVH metric for the PTV and that “High Sensitivity” might have better accuracy for DVH metrics for the rectum and bladder. We must be willing to tolerate this residual error in clinical care.

PACS number: 87.55Qr

## I. INTRODUCTION

Highly conformal radiation therapy, such as intensity‐modulated radiotherapy (IMRT) and volumetric‐modulated arc radiotherapy (VMAT), improves conformance of the dose distribution to the PTV and also reduces the impact on organ at risk (OAR). These technologies provide complex dose distributions with a sharp gradient, and patient‐specific quality assurance (QA) is therefore necessary. Commonly, point dose measurement using an ion chamber, as well as planar dose measurement using radiographic film, was traditionally implemented for dosimetric QA of treatment plans. Recently, various ion chamber or diode detector arrays have become commercially available, allowing pretreatment absolute dose verification with near real‐time results.^(1)^ Commercially available devices include ArcCHECK (Sun Nuclear Corporation, Melbourne, FL), Delta^4^ (ScandiDos, Inc., Ashland, VA), MatriXX (IBA Dosimetry GmbH, Schwarzenbruck, Germany), and Array seven29 (PTW, Freiburg, Germany).

Gamma index evaluation has become a standard technique used to compare measured distributions with calculated distributions by a commercial radiation treatment planning system (TPS).^(2)^ A typical example of an acceptance criterion of 95% of points above a dose threshold must have a gamma index less than one for dose difference and distance‐to‐agreement limits of 3% and 3 mm, respectively. A previous study^(3)^ demonstrated a lack of correlation between conventional IMRT QA methods and dose errors in anatomic regions of interest. Zhen et al.^(4)^ also reported that the gamma passing rate has a weak correlation to critical patient dose‐volume histogram (DVH) errors.

Several commercially available software systems using measurement data aiming to provide DVH‐based QA metrics have recently been developed to tackle the problem. One of these software systems is 3DVH, which can reconstruct 3D patient dose with measurement data by an ArcCHECK 3D diode array (both from Sun Nuclear Corporation). While the accuracy of 3DVH has already been investigated in several studies,^4^, ^5^, ^6^, ^7^, ^8^, ^9^ there has been no published work on clarification of the correlation between the accuracy of dose estimation with 3DVH software and MLC misalignments.

CT image sets were not required for the 3DVH calculation, and a relative 3D dose grid for each subbeam was independently calculated by convolving a 3D impulse TERMA function throughout the phantom volume with the 3D scatter depth kernels in 3DVH.^(10)^ Thus, the presence of heterogeneity would introduce errors. To minimize the effect of inhomogeneity for dose estimation calculated by 3DVH, we studied prostate VMAT plan.

The purpose of this study was to evaluate the accuracy of dosimetric parameters reconstructed by 3DVH software according to MLC position error in prostate VMAT plans.

## II. MATERIALS AND METHODS

### A. 3DVH with ArcCHECK

The commercially available diode array used in this study was ArcCHECK. It is a cylindrical 3D diode array detector and contains 1386 diodes (detector size: $0.8\times 0.8\;{\text{mm}}^{2}$) in a helical arrangement at intervals of 10 mm and with a diameter of 21 cm. The physical depth of each diode is 2.9 cm. ArcCHECK was calibrated with an absolute dose of 2 Gy with a $10\times 10\;{\text{cm}}^{2}$ field size at a gantry angle 0° before this study. To reconstruct 3D patient dose, 3DVH software version 3.0 was used. ArcCHECK Planned Dose Perturbation (ACPDP) was used to estimate a 3D patient dose by measurement‐guided dose reconstruction (MGDR). In ACPDP, perturbation of TPS‐calculated dose was done beam‐by‐beam, on a voxel‐by‐voxel basis, using correction factors per beamlet garnered from per‐beam dose planes normal to the central axis. This algorithm required that the air cavity in ArcCHECK was filled with a PMMA plug during irradiation. Further details on ACPDP have been described elsewhere.^(7)^

In this study, two different calculating modes of ACPDP reconstruction were used: “High Sensitivity” and “Normal Sensitivity”. The difference between the two relied on integrated technology and logic that detects 4D dose gradients during reconstruction and allows customization of the sensitivity of dose morphing (with respect to the helical diode matrix) in those gradients. In “Normal Sensitivity” dose morphing, there are two conditions in which the morphing may be dampened per diode: 1) if the diode's dose is below a qualifying threshold dose, and 2) the diode is in a very high‐gradient region. On the other hand, “High Sensitivity” allows a high range of dose morphing even in steep dose gradients for the 4D subbeams and in high‐ and low‐dose regions. This option is recommended for detecting even very small deviations from ideal behavior. The reconstructed dose grid size was the same as dose calculation grid size.

### B. Patient characteristics

Ten prostate cancer patients treated with hypofractionated VMAT (67.6 Gy/26 Fr) in our hospital were studied. All plans contained one or two full arcs. Each plan was generated in Eclipse treatment planning system version 8.6 (Varian Medical Systems, Palo Alto, CA). All prostate plans were planned for a 15 MV X‐ray beam on Varian 23 EX with a millennium 120 multileaf collimator. Average X jaw size with a standard deviation was 8.84±0.59 cm. Average Y jaw size with a standard deviation was 8.90±0.83 cm. The dose calculation algorithm was anisotropic analytical algorithm (AAA) version 11.0.1. The calculation grid size was $1\times 1\times 1\;{\text{mm}}^{3}$.

### C. MLC errors

**Figure 1:**
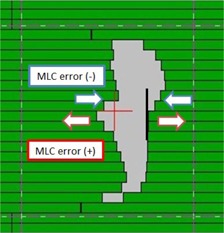
Creation of error‐induced plan. Positive errors were created by moving the MLC banks in the opposite direction that resulted in opening of MLC apertures, whereas negative errors were created by moving the MLC banks in the opposite direction that resulted in closing of MLC apertures.

Error‐induced plans were generated by in‐house software programmed by C++. The software program allowed the user to import a DICOM plan and modify the position of entire leaf banks systemically. This MLC editor was based on the study by Oliver et al..^(11)^ The MLC position errors were −0.75,−0.5,−0.25,0.25,0.5, and 0.75 mm for each single bank. Positive errors were created by moving the MLC banks in the direction that resulted in opening of MLC apertures, whereas negative errors were created by moving the MLC banks in the direction that resulted in closing of MLC apertures (Fig. 1). This software was used to simulate the investigated misalignments of the MLCs that could be applied directly to the exported DICOM plans. After MLC position errors had been introduced into the baseline plans, they were reimported into Eclipse for dose calculation and ArcCHECK plans were generated and measured. In total, seven plans and calculated dose (one baseline plan plus six different MLC error‐induced plans) were generated per patient.

### D. Comparison of calculated and estimated 3D patient doses

**Figure 2:**
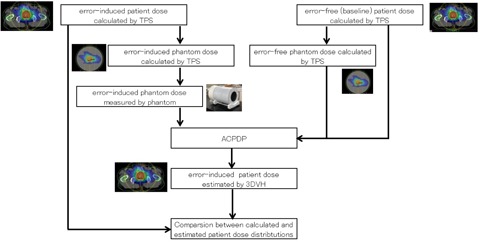
A schematic diagram for comparison of calculated 3D patient dose and estimated 3D patient dose. Error‐induced patient dose was estimated by 3DVH using error‐induced phantom dose measured by a phantom, error‐free phantom dose calculated by TPS, and error‐free patient dose calculated by TPS. Then we evaluated the differences in QA metrics between the calculated and estimated 3D patient dose.

**Figure 3:**
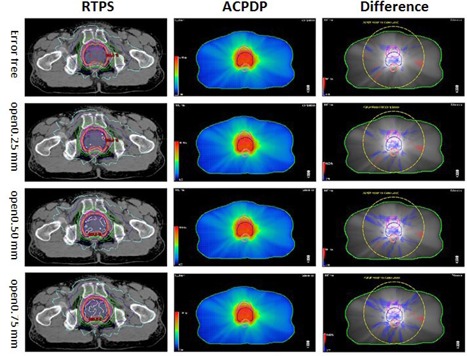
Schematic design of comparison between TPS dose and ACPDP dose. The TPS dose and the ACPDP dose shown in this figure are: (a) baseline dose, (b) +0.25 mm MLC position, (c) +0.5 mm MLC position, and (d) +0.75 mm MLC position. The different figure shown on the right was created by results of gamma analysis (criteria: 1%/1 mm, TH=10% of global max. dose).

Figure 2 shows a flow chart of comparison between 3D patient dose calculated by TPS and that estimated by 3DVH using the error‐induced plan. First, we calculated 3D patient dose reconstructed by the ACPDP algorithm (ACPDP dose) using the error‐induced measured dose and the baseline plan calculated by TPS. Then, as shown in the schematic design of Fig. 3, we evaluated the differences between ACPDP dose with error and calculated 3D patient dose with error by TPS (TPS dose). The dose estimation error was calculated as described in Eq. (1)

(1) $$Dose\;estimation\;error=\frac{D_{3DVH}-D_{TPS}}{D_{TPS}}\times 100$$

where $D_{3DVH}$ is ACPDP dose calculated by 3DVH using measurement of error‐induced plan and TPS dose of error‐free plan, and $D_{TPS}$ is TPS dose of error‐induced plan.

### E. Data analysis: DVH‐based metrics and gamma analysis

The correlation between accuracy of estimation and MLC position error was evaluated. In this study, data analysis was done in terms of two metrics: DVH‐based metrics and the gamma analysis. DVH‐based metrics was done in 3DVH software with the following structures: for PTV (mean dose and D95) and for rectum and bladder walls (mean dose, V35, and V55). ACPDP dose and TPS dose were compared by these methods using global gamma analysis with different criteria (3%/3 mm, 2%2 mm, and 1%/1 mm), and the lower threshold for the gamma analysis was 10% of the global maximum dose. The difference in dose estimation error between “Normal Sensitivity” and “High Sensitivity” was compared using a Wilcoxon test with JMP version 11 (SAS Institute, Cary, NC).

## III. RESULTS

### A. Comparison of gamma passing rate between 2D planar dose measured by ArcCHECK vs. TPS dose in baseline plan

Before using 3DVH, we evaluated the planar dose measured by ArcCHECK using an SNC Patient version 6.2.3 (Sun Nuclear Corporation) for 2D gamma analysis. Table 1 shows the global gamma passing rates between measured planar dose and calculated planar dose in the baseline plan, indicating good agreement between two dose distributions with gamma passing rate >95% for all patients (3 mm/3%).

**Table 1:** Global gamma passing rates between measured planar dose and calculated planar dose in the baseline plan.

	*Global Gamma Passing Rate (%)*		
*Patient*	1%/1 mm	2%/2 mm	3%/3 mm
1	77.9	98.2	99.6
2	74.8	95.4	99.3
3	75.7	97.4	99.6
4	75.8	95.5	99
5	81.9	98.9	100
6	79.3	97.4	99.6
7	80.3	99.1	99.8
8	80.3	97.3	99.3
9	86	99.2	100
10	83.6	98.6	100
Average	81.0	98.5	99.9
SD	4.1	1.0	0.2

### B. Dose percent change between baseline and error‐induced plans

**Figure 4:**
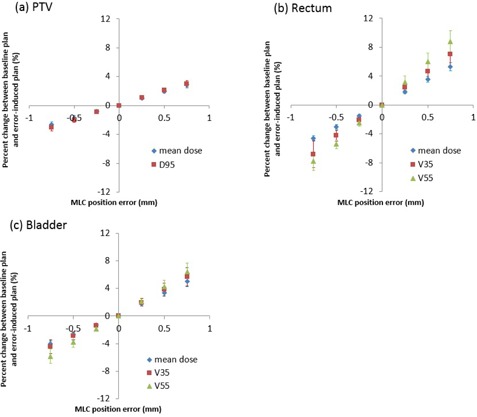
Average change between error‐free (baseline) and error‐induced plans for QA metrics in (a) PTV, (b) rectum, and (c) bladder.

Figure 4 shows the percent dose changes between baseline TPS dose and error‐induced TPS dose in each DVH metric. The percent dose change linearly increased with increase in the MLC position error. For mean dose of PTV, rectum, and bladder, the percent changes with −0.75 mm MLC error were −2.67%,−4.61%, and −4.06%, respectively, whereas those with +0.75 mm were 2.90%, 5.26%, and 5.02%, respectively. Correlation coefficient determination $\left(R^{2}\right)$ for mean dose for PTV, rectum, and bladder were 0.9995, 0.9990, and 0.9973, respectively. $R^{2}$ for V35 for the rectum and bladder and V55 for the rectum and bladder were 0.9996, 0.9962, 0.9990, and 0.9994, respectively. These results indicated a strong positive linear correlation.

### C. Correlation between dose estimation error and MLC position error using gamma analysis

**Figure 5:**
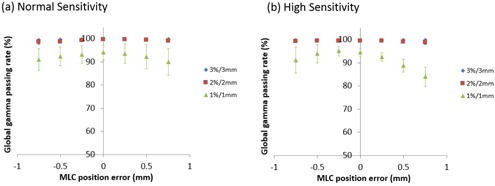
Correlation between global gamma passing rate and MLC position error for “Normal Sensitivity” and “High Sensitivity” on average for all ten patients.

We calculated the global gamma passing rate using three different criteria (3%/3 mm, 2%/2 mm, and 1%/1 mm) for “Normal Sensitivity” and “High Sensitivity”. Figure 5 shows the correlation between global gamma passing rate and MLC position error on average for all patients. A comparison using 1%/1 mm, the most stringent criterion in this study, showed that the larger was the MLC error induced, the worse were the gamma passing rates (for example, in “High Sensitivity”, global gamma passing rates changed from 94.5% at baseline down to 84.0% at +0.75 mm error). Besides, “High Sensitivity” had a larger fall in gamma passing rate than did “Normal Sensitivity”, according to MLC position error. That is, 3DVH described the induced dose errors more accurately with “Normal Sensitivity” than with “High Sensitivity”. On the other hand, the 2%/2 mm and 3%/3 mm gamma passing rates show the nearly identical gamma passing rates (>98%) for all plans in “Normal” and “High Sensitivity”. This result showed the estimation error was too small to capture the error by the 2%/2 mm and 3%/3 mm gamma passing rates. There are no significant difference between “Normal Sensitivity” and “High Sensitivity” for all gamma passing rates (1%/1 mm:p=0.44,2%/2 mm:p=0.73,3%/3 mm:p=0.15).

### D. Correlation between dose estimation error and MLC position error for DVH metric

6, 7 show correlations between dose estimation error of each DVH metric and MLC position error for all ten patients using “Normal Sensitivity” and “High Sensitivity”. The dose estimation error shown on the y‐axis was the percent dose difference between the 3DVH dose with error and the TPS dose with error as described in Eq. (1). This meant that 3DVH underestimated the impact of the induced error when the dose estimation error was a negative value and that 3DVH overestimated it when the dose estimation error was a positive value.

**Figure 6:**
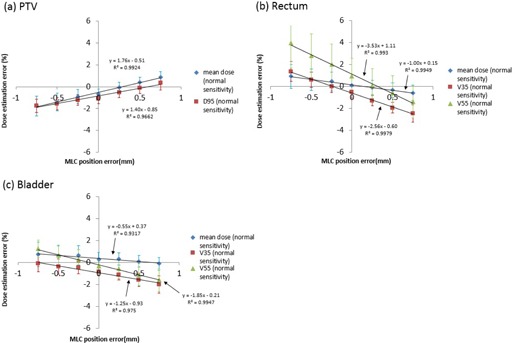
Correlation between dose estimation error and MLC position error in (a) PTV, (b) rectum, and (c) bladder for “Normal Sensitivity” on average for all ten patients.

**Figure 7:**
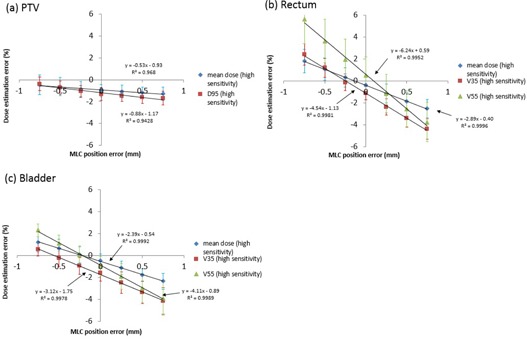
Correlation of dose estimation error and MLC position error in (a) PTV, (b) rectum, and (c) bladder for “High Sensitivity” on average for all ten patients.

For the baseline plan, small dose estimation errors (<2.0%) were seen for all DVH metrics with both sensitivities. For error‐induced plans, 3DVH estimation accuracy for all DVH metrics varied with MLC position errors (especially V35 and V55 for the rectum), indicating that 3DVH has some residual error in clinical care.

6, 7 also report the slope, y‐intercept, and $R^{2}$ for each linear fit to the dose estimation error versus MLC position error. Slopes of linear fit to dose estimation error versus MLC position error for mean dose and D95 to the PTV were 1.76 and $1.40\%\;{\text{mm}}^{-1}$, respectively, for “Normal Sensitivity”, and −0.53 and $-0.88\%\;{\text{mm}}^{-1}$, respectively, for “High Sensitivity”, showing better accuracy for “High Sensitivity” than “Normal Sensitivity”. On the other hand, the slopes for mean dose to the rectum and bladder, V35 to the rectum and bladder, and V55 to the rectum and bladder were −1.00, −0.55, −2.56, −1.25, −3.53, and $-1.85\%\;{\text{mm}}^{-1}$, respectively, for “Normal Sensitivity”, and −2.89, −2.39, −4.54, −3.12, −6.24, and $-4.11\%\;{\text{mm}}^{-1}$, respectively, for “High Sensitivity“, showing significant better accuracy for “Normal Sensitivity” than “High Sensitivity”. For all dosimetric parameters, except for mean dose and D95 to the PTV with “Normal Sensitivity”, there was a negative correlation between dose estimation error and MLC position error. All $R^{2}$ for all dosimetric parameters were more than 0.94. These results demonstrated that the dose estimation error had a strong correlation with MLC position error. For almost all parameters, the estimation error tended to become a negative value with increase of the opening MLC position error and it tended to become a positive value with increase of the closing MLC position error, suggesting that 3DVH might do a weak perturbation (correlation) of error‐free TPS dose.

To investigate how much the induced error was captured by 3DVH, we did an additional analysis of the ratio of 3DVH estimation error (shown in 6, 7) to percent dose change (shown in Fig. 4) as shown in Eq. (2). In the calculation, it was assumed that the 3DVH estimation error for the baseline plan was zero.

(2) $$R_{x}=\left(1-\left|\frac{3DVH_{x}-3DVH_{0}}{D_{x}}\right|\right)$$

where $R_{x}$ is the ratio of 3DVH estimation error with x mm MLC position error $\left(3DVH_{x}\right)$ to percent dose change from the baseline plan to the error‐induced plan with x mm MLC position error ($\left(D_{x}\right)$. The average ratios for DVH metrics in the PTV, rectum, and bladder for all patients are summarized in Table 2. The ratios for DVH metrics in the rectum and bladder were more than 0.55 (range: 0.57–0.88) and those in the PTV were more than 0.4 (range: 0.41–0.74) for “Normal Sensitivity”, whereas those in the rectum and bladder were less than 0.3 (range: 0.32–0.63) and those in the PTV were more than 0.7 (range: 0.71–0.91), indicating that 3DVH could capture a part of the induced error but not all of the induced error. Furthermore, these results indicated that “Normal Sensitivity” might have better accuracy for the DVH metric for the PTV and that “High Sensitivity” might have better accuracy for DVH metrics for the rectum and bladder.

**Table 2:** Summary of average ratios of 3DVH estimation error to percent dose change from baseline and error‐induced plan for each DVH metric in PTV, rectum, and bladder for ten patients (mean±SD).

*MLC Position Error (mm)*		*Normal Sensitivity*					
		−*0.75*	−*0.5*	−*0.25*	*0.25*	*0.5*	*0.75*
PTV	Mean dose	0.56±0.29	0.52±0.37	0.49±0.36	0.41±0.18	0.44±0.14	0.46±0.15
	D95	0.74±0.21	0.67±0.27	0.55±0.29	0.50±0.22	0.51±0.15	0.51±0.18
Rectum	Mean dose	0.82±0.11	0.81±0.10	0.75±0.18	0.88±0.08	0.88±0.08	0.87±0.09
	V35	0.71±0.11	0.74±0.12	0.73±0.19	0.62±0.30	0.68±0.18	0.70±0.15
	V55	0.59±0.20	0.66±0.10	0.57±0.21	0.71±0.11	0.74±0.08	0.73±0.08
Bladder	Mean dose	0.85±0.12	0.79±0.16	0.66±0.41	0.83±0.16	0.88±0.08	0.88±0.08
	V35	0.80±0.12	0.77±0.12	0.72±0.22	0.80±0.12	0.79±0.14	0.78±0.15
	V55	0.73±0.09	0.74±0.11	0.68±0.20	0.88±0.10	0.82±0.05	0.80±0.05
		*High Sensitivity*					
PTV	Mean dose	0.81±0.14	0.78±0.16	0.73±0.15	0.85±0.10	0.90±0.08	0.91±0.07
	D95	0.71±0.13	0.72±0.18	0.73±0.18	0.79±0.12	0.86±0.09	0.88±0.06
Rectum	Mean dose	0.53±0.06	0.53±0.09	0.53±0.14	0.58±0.12	0.58±0.06	0.60±0.05
	V35	0.46±0.10	0.45±0.10	0.51±0.14	0.49±0.10	0.51±0.06	0.53±0.06
	V55	0.32±0.20	0.42±0.10	0.39±0.24	0.50±0.11	0.49±0.07	0.52±0.06
Bladder	Mean dose	0.59±0.09	0.59±0.13	0.60±0.16	0.63±0.10	0.62±0.03	0.63±0.04
	V35	0.50±0.09	0.51±0.13	0.53±0.15	0.56±0.06	0.54±0.06	0.55±0.08
	V55	0.44±0.06	0.45±0.09	0.45±0.10	0.57±0.16	0.54±0.06	0.54±0.04

## IV. DISCUSSION

Traditional patient‐specific QA is based on calculated 2D gamma analysis. Current developments indicate that the 2D gamma may not be able to detect small, but clinically significant, variations between the plan and actually delivered dose.^(12)^ In this study, we investigated the correlations of 3DVH accuracy and MLC position error in prostate VMAT plans. A comparison using 1%/1 mm showed that the larger was the MLC error induced, the worse were the gamma passing rates (for example, in “High Sensitivity”, global gamma passing rates changed from 94.5% at baseline down to 84.0% at +0.75 mm error). The 3DVH estimation accuracy for all DVH metrics varied with MLC position errors (especially V35 and V55 for rectum) and 3DVH could capture a part of induced error, but not all of induced error, showing that the 3DVH had more or less residual error.

For correlations between 3DVH accuracy and MLC position error, Fig. 5 shows a fall in gamma‐passing rate with the criteria 1%/1 mm, especially “High Sensitivity”, indicating that the larger was the MLC error induced, the worse was the 3DVH accuracy. 6, 7 also show correlations between 3DVH accuracy and MLC position error for each DVH metric for PTV, rectum, and bladder. These results indicated that the correlations varied with each structure and metric. A positive error tended to cause underestimation of 3DVH and a negative error tended to cause overestimation of 3DVH for both sensitivities. That is, 3DVH might do a weak perturbation (correlation) to error‐free TPS dose.

For comparison of two different sensitivities, Fig. 5 shows that “High Sensitivity” had a larger fall in gamma passing rate than did “Normal Sensitivity” according to MLC position error. 6, 7 and Table 2 show that the 3DVH described the induced dose errors more accurately for PTV with “High Sensitivity” than that with “Normal Sensitivity”. On the other hand, 3DVH described the induced dose errors more accurately for the rectum and bladder with “Normal Sensitivity” than that with “High Sensitivity”. The dose morphing with “Normal Sensitivity” might be dampened in high‐gradient region. On the other hand, “High Sensitivity” allows a high range of dose morphing even in steep dose gradients. As the dose to the PTV was within high dose or steep dose gradient regions, “High Sensitivity” might be able to morph the dose accurately. However, “High Sensitivity” might cause the excessive dose morphing, resulting in less accuracy outside the PTV (rectum and bladder) and lower gamma passing with criteria 1%/1 mm.

For dose estimation accuracy for baseline plan (not error‐induced plan), although the dose difference between 3DVH dose and TPS dose should be zero ideally, there were small dose differences in gamma‐passing rate (Table 1) (approximately 80% at 1%/1 mm) and DVH‐based parameters (6, 7) (approximately 1.0%–1.5%). The similar dose difference between 3DVH and TPS was reported by previous papers. Watanabe and Nakaguchi^(5)^ reported that the mean PTV dose difference between 3DVH and TPS was −0.6%. Stasi et al.^(6)^ showed that mean dose differences between 3DVH and TPS dose for mean dose to PTV, V70 for rectum were −1.78% and −1.31%, respectively. This result is consistent with our results. The reason for this might be due to systematic issue in the beam modeling of TPS and the uncertainty in the beam model of ACPDP model.^(13)^

## V. CONCLUSIONS

We investigated the correlations of 3DVH accuracy and MLC position error in prostate VMAT plans for ten patients. A comparison using 1%/1 mm showed that the larger was the MLC error‐induced, the worse were the gamma passing rates. The 3DVH estimation accuracy for all DVH metrics varied with MLC position errors (especially V35 and V55 for rectum) and 3DVH could capture a part of induced error, but not all of induced error, showing that the 3DVH had some error. Furthermore, we found that “Normal Sensitivity” might have better accuracy for the DVH metric for the PTV and that “High Sensitivity” might have better accuracy for DVH metrics for the rectum and bladder. We must be willing to tolerate this residual error in clinical care.

## ACKNOWLEDGMENTS

Sun Nuclear Corporation (Melbourne, FL, USA) and TOYO MEDIC Corporation (Tokyo, Japan) are gratefully acknowledged for providing the detailed information of 3DVH software.
